# Incorporating ventilatory support parameters into the PaO_2_/FiO_2_ ratio in ARDS patients

**DOI:** 10.1186/s13613-021-00926-1

**Published:** 2021-09-22

**Authors:** Mohamad F. El-Khatib, Imad J. BouAkl, Ali H. Hallal

**Affiliations:** 1 Department of Anesthesiology, American University of Beirut, Beirut, Lebanon; 2 Department of Internal Medicine, American University of Beirut, Beirut, Lebanon; 3 Department of Surgery, American University of Beirut, Beirut, Lebanon

To the Editor,

We read with great interest the study by Palanidurai et al. in which the authors compare the predictive validity for hospital mortality of a new oxygenation index (P/FP) (P/FP = 10 × PaO_2_/(FiO_2_ × PEEP) versus the classical P/F ratio and evaluate changes in severity classification of ARDS from the use of the P/FP rather than the P/F ratio [1]. We totally concur with the notion that the P/F ratio can be significantly improved for superior reflection of ARDS severity and prediction of intensive care unit survival/mortality by incorporating mechanical ventilatory support variables into the P/F ratio. The authors argued that “in general for the same P/F ratio, a patient on a higher PEEP has more severe ARDS than a patient on a lower PEEP” which makes perfect sense. For that reason, they elected to incorporate PEEP to the P/F ratio. However, we can further argue that for the same P/F ratio, a patient on a higher mean airway pressure ($\overline{{{\text{Paw}}}}$) has more severe ARDS than a patient on a lower $\overline{{{\text{Paw}}}}$ even when PEEP levels are the same or even irrespective of the PEEP levels. $\overline{{{\text{Paw}}}}$ not only reflects the applied PEEP during mechanical ventilation but also takes into consideration other important mechanical ventilatory support variables such as inspiration:expiration ratio (I:E) and peak inspiratory and alveolar pressures [2].

Previously, we have described a new oxygenation index, termed oxygenation factor (OF) that incorporates $\overline{{{\text{Paw}}}}$ and is expressed as:

$${\text{OF}} = \frac{{{\text{P/F}}}}{{\overline{{{\text{Paw}}}} }} = \frac{{{\text{P}}_{{\text{a}}} {\text{O}}_{2} }}{{{\text{F}}_{{\text{i}}} {\text{O}}_{2} \times \overline{{{\text{Paw}}}} }}.$$

As such, the oxygenation factor (OF) will normalize the P/F ratio to the mean airway pressure. Our study showed that the OF is more reliable than the P/F ratio in reflecting intrapulmonary shunt in patients undergoing coronary artery bypass grafting and with no underlying lung diseases [3]. Recently, we published a study in which we compared our oxygenation (OF) to the P/F ratio in 50 ARDS patients with P/F ≤ 100 mm Hg, 50 ARDS patients with 100 mm Hg < P/F ≤ 200 mm Hg, and 50 ARDS patients with 200 < OR ≤ 300 mm Hg. Our results showed that the OF is superior to the P/F ratio in reflecting oxygenation in ARDS and results in a different patients’ classification for ARDS severity [3].

The study by Palanidurai et al. incorporated only PEEP in the P/F ratio and overlooked other important mechanical ventilatory support parameters such as I:E ratio, and peak inspiratory and alveolar pressures and subsequently the driving pressure. The advantage of incorporating the mean airway pressure ($\overline{{{\text{Paw}}}}$) rather than PEEP into the P/F ratio such as in our OF index is that $\overline{{{\text{Paw}}}}$ can be considered a more appropriate modifier for the P/F ratio since it better reflects the mean alveolar pressure and as such reflects not only the applied PEEP but rather most of the mechanical ventilation parameters and settings such as I:E ratio, tidal volume, and/or peak alveolar and driving pressures [4]. Another concern with the Palanidurai et al. P/FP ratio is the use of a correction factor of 10. Although the authors provided several justifications for this approach, it remains unclear why the authors did not keep their P/FP index as is [i.e., P/FP = PaO_2_/(FiO_2_ × PEEP)] and proceeded to assess and evaluate their index for superior reflection of ARDS severity and prediction of survival/mortality without the use of the correction factor. Furthermore, Palanidurai et al. used the Berlin definition’s thresholds of ≤ 100, 101–200, and 201–300 to differentiate severe, moderate and mild ARDS, respectively, for both the P/F (mmHg) and the P/FP (mmHg/cmH_2_O) ratios. This makes sense only when PEEP is 10 cmH_2_O because in such case the P/FP ratio will always be equal to P/F ratio due to the presence of the correction factor 10. What about ARDS patients with different levels of PEEP (i.e., PEEP is not 10 cmH_2_O) and will the same thresholds applied in the Berlin definition remain applicable for the new P/FP ratio.

Nevertheless, Palanidurai et al. deserve to be commended for conducting such a valuable study and providing data on a highly needed subject [1]. Both their results and our recently reported results clearly support the concept that multifactorial oxygenation indexes such as the P/FP and OF ratios that incorporate mechanical ventilatory support parameters into the classical P/F ratio are superior indexes to use in mechanically ventilated ARDS patients, particularly for assessing the oxygenation status and the classification of ARDS severity and that these indexes have a greater predictive validity for hospital mortality in ARDS than the P/F ratio [1, 4]. In ARDS patients, oxygenation indexes should be multifactorial and should reflect not only the true illness severity, but also the applied mechanical ventilation strategies.

## Data Availability

Not applicable.
